# Genetic and functional characterization of putative Ras/Raf interaction inhibitors in *C. elegans *and mammalian cells

**DOI:** 10.1186/1750-2187-5-2

**Published:** 2010-02-23

**Authors:** Vanessa González-Pérez, David J Reiner, Jamie K Alan, Cicely Mitchell, Lloyd J Edwards, Vladimir Khazak, Channing J Der, Adrienne D Cox

**Affiliations:** 1 Curriculum in Genetics and Molecular Biology, University of North Carolina at Chapel Hill, Chapel Hill, NC, 27599, USA; 2 Lineberger Comprehensive Cancer Center, University of North Carolina at Chapel Hill, Chapel Hill, NC, 27599, USA; 3 Department of Pharmacology, University of North Carolina at Chapel Hill, Chapel Hill, NC, 27599, USA; 4 Department of Biostatistics, University of North Carolina at Chapel Hill, Chapel Hill, NC, 27599, USA; 5 NexusPharma, Inc., 253-13 Summit Sq. Ctr., Langhorne, PA, 19047, USA; 6 Department of Radiation Oncology, University of North Carolina at Chapel Hill, Chapel Hill, NC, 27599, USA

## Abstract

**Background:**

Activation of the mammalian Ras-Raf-MEK-ERK MAPK signaling cascade promotes cellular proliferation, and activating Ras mutations are implicated in cancer onset and maintenance. This pathway, a therapeutic target of interest, is highly conserved and required for vulval development in *C. elegans*. Gain-of-function mutations in the Ras ortholog lead to constitutive pathway signaling and a multivulva (Muv) phenotype. MCP compounds were identified in a yeast two-hybrid screen for their ability to disrupt Ras-Raf interactions. However, this had not been confirmed in another system, and conflicting results were reported regarding selective MCP-mediated blockade of Ras- and Raf-mediated biological activities in mammalian cells. Here we used the easily-scored Muv phenotype as an *in vivo *readout to characterize the selectivity of MCP110 and its analogs, and performed biochemical studies in mammalian cells to determine whether MCP treatment results in impaired interaction between Ras and its effector Raf.

**Results:**

Our genetic analyses showed significant dose-dependent MCP-mediated reduction of Muv in *C. elegans *strains with activating mutations in orthologs of Ras (LET-60) or Raf (LIN-45), but not MAP kinases or an Ets-like transcription factor. Thus, these inhibitors selectively impair pathway function downstream of Ras and upstream of or at the level of Raf, consistent with disruption of the Ras/Raf interaction. Our biochemical analyses of MCP110-mediated disruption of Ras-Raf interactions in mammalian cells showed that MCP110 dose-dependently reduced Raf-RBD pulldown of Ras, displaced a fluorescently-tagged Raf-RBD probe from plasma membrane locations of active Ras to the cytosol and other compartments, and decreased active, phosphorylated ERK1/2.

**Conclusions:**

We have effectively utilized *C. elegans *as an *in vivo *genetic system to evaluate the activity and selectivity of inhibitors intended to target the Ras-Raf-MAPK pathway. We demonstrated the ability of MCP110 to disrupt, at the level of Ras/Raf, the Muv phenotype induced by chronic activation of this pathway in *C. elegans*. In mammalian cells, we not only demonstrated MCP-mediated blockade of the physical interaction between Ras and Raf, but also narrowed the site of interaction on Raf to the RBD, and showed consequent functional impairment of the Ras-Raf-MEK-ERK pathway in both *in vivo *and cell-based systems.

## Background

Over the past two decades, there have been many attempts to isolate and characterize pharmacological inhibitors targeting Ras-dependent signaling pathways. The small GTPase Ras normally transmits signals downstream of diverse inputs and is a critical signaling node for many cellular activities. Aberrant Ras activity leads to the deregulation of numerous cellular processes including proliferation, survival, cell adhesion and migration, that in turn can contribute to cellular transformation, invasion and metastasis [1], and Ras is mutationally activated in ~30% of cancers [2]. Among the downstream effectors of Ras, the most well-characterized is the Ras-Raf-MAPK signaling pathway, in which Ras interaction with the serine/threonine kinase Raf causes a cascade of kinase activation, with Raf activating the mitogen-activated protein kinase kinases (MAPKK, or MEK) and MEK activating the ERK MAPK, which then translocates to the nucleus to phosphorylate and activate transcription factors to carry out the commands of Ras. The B-Raf isoform is mutationally activated, most commonly at V600E, in tumors including colorectal cancer, malignant melanoma and thyroid cancer [3,4], in a manner mutually exclusive with oncogenic Ras. Aberrant activation of MAPK has also been associated with various cancers [5]. Given the relevance of the Ras-Raf-MAPK signaling pathway to a wide array of malignancies, there has been a great deal of interest in developing anti-cancer therapeutics by targeting specific elements of this pathway [6-9]. Despite intensive efforts [10], it has proven very difficult to selectively target Ras itself, which at present is widely viewed as "undruggable" due to the picomolar affinity of GTP for Ras. Pharmacological inhibition of the Raf and MEK kinases has been seen as more tractable, and several putative Raf inhibitors have reached clinical trials, including both antisense and kinase inhibitors. The most prominent of these, BAY43-9006 (sorafenib), was originally described as a Raf kinase inhibitor [11,12], but its activity in cancer patients did not correlate with Raf activation or mutational status. Instead, it demonstrated additional activity towards the pro-angiogenic vascular endothelial growth factor receptors (VEGFR)-2 and -3, and to other receptor tyrosine kinases such as PDGFR-beta that are also involved in tumorigenesis [13,14]. Thus, the anti-tumor effects of sorafenib, now known as a "multikinase inhibitor", are at least partly mediated by blockade of VEGFR kinase rather than Raf kinase. Newer Raf kinase inhibitors such as PLX4032 [15] and its later derivatives, intended to be selective for mutationally activated B-Raf (V600E), are also under development [16]. Extensive investment has also been made in MEK inhibitors including CI-1040, AZD6244 and others [6,8,9,17], although none has yet proven efficacious as single agent therapy.

Another approach to inhibit the Ras-Raf-MAPK signaling pathway is through protein-protein interaction (PPI) inhibitors such as those intended to disrupt the interaction between the small GTPase Ras and the serine/threonine kinase Raf [6]. Here, we characterized the activity of a novel family of putative Ras/Raf interaction inhibitors derived from such a search. MCP compounds such as MCP1 were originally isolated from a small molecule library using a dual-bait two-hybrid system to probe the interaction between Ras and Raf [18-20]. The more advanced MCP110 and MCP116 as well as a very weakly active analog MCP122 were synthesized during optimization efforts. Earlier reports characterizing the activity of these agents showed their ability to inhibit Ras signaling and Ras-mediated cell proliferation and anchorage-independent growth in cell-based systems, as well as transformed growth in nude mouse xenografts [20-22]. However, the mechanism of action of these putative Ras/Raf interaction inhibitors is not completely understood. The ability of MCP1 and later analogs such as MCP110 to inhibit Ras- but not Raf-mediated transformation in fibroblasts, colorectal cancer cell lines and melanoma cell lines suggested that their action was at the level of Ras rather than Raf, but more recent evidence indicated that MCP compounds also have activity towards melanoma cell lines in which B-Raf is mutated [23]. Therefore it is unclear whether the anti-transformation activity of MCP compounds is due to blocking Ras, Raf or yet another target. Whether MCP compounds directly disrupt the physical interaction between Ras and Raf, as shown by the yeast two-hybrid assay in which they were originally identified, has not been confirmed in mammalian cells.

Characterizing the precise mechanism of action of PPI inhibitors is a challenging task, especially given the difficulty of determining whether a given compound is interacting with the interface of one protein versus the other. There are no structural analyses available to reveal whether MCP compounds bind physically to Ras, to Raf, or to both. We therefore set out to determine at what level in the pathway MCP compounds act, by using epistasis analyses in the nematode *Caenorhabditis elegans*, a tractable genetic model system for the *in vivo *evaluation of Ras pathway drug activity.

*C. elegans *has served as a very useful model organism to study development, neurobiology and many other biological processes. Recently it has also been useful in pharmacogenetic studies to identify the targets of pharmacological agents [24]. The *C. elegans *Ras-Raf-MAPK signaling pathway is highly conserved, from the EGF ligand to the transcriptional output [25,26]. LET-60, the *C. elegans *ortholog of Ras, is critical to regulate vulval development [27], and excessive activation at any level of the pathway results in hyperinduction of vulval tissue, leading to a Multivulva (Muv) phenotype. For example, a glycine to glutamic acid mutation at residue 13 (G13E) of LET-60/Ras, results in a gain-of-function that produces a constitutively activated LET-60 protein, analogous to the well known Ras(G12V) mutation in mammalian cells. Not surprisingly, then, LET-60(G13E) is well documented to induce the Muv phenotype [27], as do transgenes bearing activated Raf (LIN-45) or MEK/MAPK (MEK-2/MPK-1) and loss-of-function mutations in the downstream Ets-like transcription factor LIN-1 [28-30]. Previous work by our group and others has validated these transgenes and the Muv phenotype of *C. elegans *as *in vivo *readouts to evaluate the activity of pharmacological inhibitors of the Ras-Raf-MEK-ERK pathway [25,31] and to identify pharmacological targets [24]. We therefore selected this system to characterize the activity and selectivity of known and novel MCP compounds.

We first confirmed that MCP110 acts downstream of Ras/LET-60 and upstream or at the level of Raf/LIN-45, as would be expected for an inhibitor of the Ras/Raf interaction. In addition, we demonstrate here that of two previously uncharacterized MCP110 analogs, MCP116, but not MCP146, also inhibits Ras/LET-60 signaling and displays specificity comparable to MCP110. Finally, for the first time we show evidence in mammalian cells that MCP110 disrupts not only signaling from Ras to ERK but also the physical interaction between Ras and Raf, and have narrowed the interface on Raf to the Ras-binding domain.

## Results

### The *C. elegans *Ras signaling pathway as a platform for analysis of small molecule inhibitors

We have previously established the multivulva (Muv) phenotype of the nematode worm *C. elegans *as an *in vivo model *system to study the action of pharmacological inhibitors targeting Ras-induced signaling cascades. Specifically, we used the well-characterized selective MEK inhibitor U0126 to demonstrate that effective pharmacological inhibition of the Ras-Raf-MEK pathways restored a normal phenotype in animals that would otherwise display a Muv phenotype based on their genetic background [25]. The Ras-Raf signal that controls vulval cell fate in *C. elegans *is well described at the molecular genetic level. Consequently, many genetic reagents, including both *in situ *mutations and transgenic constructs, are available for pharmacological dissection of the Ras pathway. In this study, we exploited activated Ras, activated Raf, combined activated MEK/ERK, and loss of an Ets transcription factor, all of which result in excessive vulval induction. For clarity we refer to these four reagents, which are described further in the Methods section, as Ras, RafAA, MEK/ERK and Ets.

In this system, wild type animals have normal vulvae accompanied by no ventral protrusions. In contrast, animals with excessive Ras pathway activity display hyperinduction of epithelial cells that results in a Muv phenotype, characterized by ectopic nonfunctional pseudovulvae that are visible as ventral protrusions. Both phenotypes can be scored under a dissecting stereomicroscope, and can be quantified either in a binary manner as Muv or non-Muv(WT), or by the number (0-3) of ectopic pseudovulvae. Thus, animals with one or more ventral protrusions are scored as Muv, whereas those with a fully developed vulva but no protrusions are scored as wild type. The consequences of drug treatment can be quantified precisely by scoring the phenotypes of animals whose vulval development proceeds in the presence of the pharmacological inhibitor [25]. Here we used this validated system to test the activity and target selectivity of small molecules that are putative Ras/Raf interaction inhibitors, MCP110 [18-20] and its novel analogs MCP116 and MCP146.

**Figure 1:**
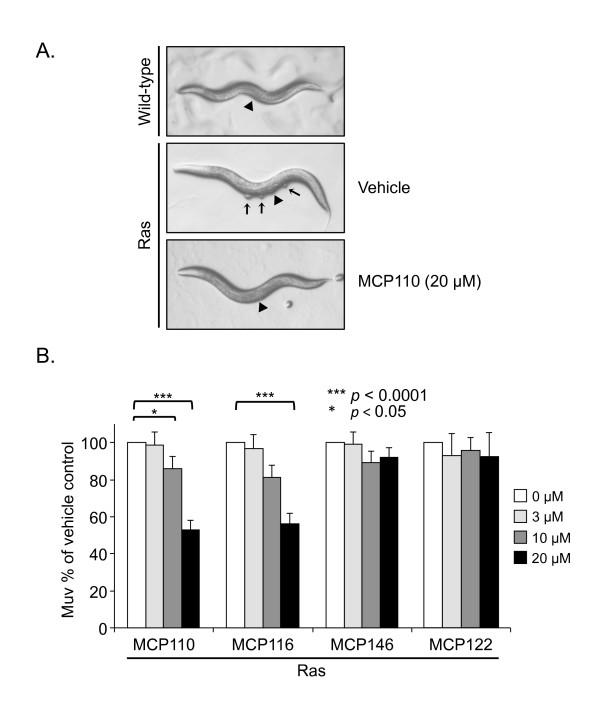
**MCP110 and MCP116 but not MCP146 inhibit the Ras/LET-60-induced Muv phenotype**. **A**. Representative images of untreated wild-type animals (WT phenotype with normal vulva (arrowhead) but no ventral protrusions); animals harboring constitutively activated Ras treated with vehicle only (Muv phenotype; arrows mark ventral protrusions formed by pseudovulvae); the same strain following treatment with MCP110 (20 μM) (WT phenotype, as indicated by lack of ventral protrusions). **B**. Animals harboring constitutively activated Ras as in Panel A were treated with either vehicle (DMSO) or MCP110, MCP116, MCP146 or negative control MCP122 (3, 10 or 20 μM; higher concentrations precipitated out of solution). The Y-axis indicates the percentage of MCP-treated animals with a Muv phenotype, normalized to vehicle-treated animals. Data were analyzed by three-way ANOVA. (***) and (*) indicate *p *values of < 0.0001 and < 0.05, respectively.

To illustrate the phenotypes described above and quantitated in our study, we show images of animals grown under different drug conditions (Figure 1A). Wild-type animals have a normal vulva (arrowhead) and an undisrupted ventral surface. Animals expressing activated Ras display the expected Muv phenotype when treated with vehicle (DMSO) only; both the functional vulva (arrowhead) and three additional ventral protrusions (arrows) are identifiable. In contrast, activated Ras animals treated with MCP110 do not display the typical Ras-induced Muv phenotype, but rather have a single properly developed vulva and no protrusions. Thus, MCP-treated Ras animals have the same appearance as wild type animals, consistent with depressed Ras-Raf-MEK-ERK signaling [25,31].

### MCP110 and MCP116, putative Ras/Raf interaction inhibitors, reverse the hyper-induced Muv phenotype conferred by activated Ras

We scored the Muv phenotype of activated Ras animals grown in the presence of MCP inhibitors or DMSO vehicle. Developmentally synchronous animals were collected from each treatment group (see Methods for details) and the Muv phenotype scored according to the presence and number of ectopic pseudovulvae. Animals displaying a Muv phenotype when drug-treated were normalized to the level of hyper-induction of Muv seen in vehicle-treated animals, with the baseline for Muv established separately for each genotype.

We expected the Muv phenotype to be sensitive to MCP compounds if the Ras-Raf interaction was successfully inhibited, and therefore that treated animals would display normal vulval development. As expected, we observed (Fig. 1B) that animals expressing activated Ras/LET-60 treated with MCP110 (20 μM) were approximately 50% less likely than vehicle-treated animals to display a Muv phenotype. Delivery to these animals of drug concentrations higher than 20 μM was not possible due to MCP compound precipitation. Additionally, we observed that the previously uncharacterized MCP110 analog, MCP116, showed inhibitory activity similar to that of MCP110. Effects of both MCP110 and MCP116 were dose-dependent. In contrast, a third derivative, MCP146, showed no significant activity at any tested concentration. As an additional negative control, we show that treatment with the poorly active analog MCP122 [20,21,23] had no effect. Together, these results indicate that both MCP110 and MCP116 inhibit the Ras-Raf-MAP kinase pathway downstream of Ras activation. This conclusion is consistent with the reported ability of MCP110 to inhibit Ras/Raf interactions in yeast and with its biological activities in mammalian cells.

### MCP compound inhibition of the Muv phenotype is specific to the Ras pathway

**Figure 2:**
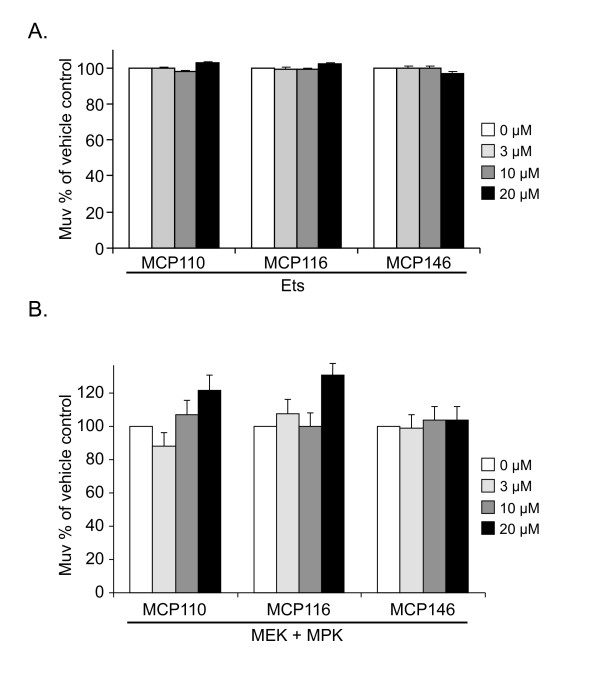
**MCP110 and derivatives do not inhibit the Muv phenotype induced by activation of the Ras-Raf-MAPK pathway downstream of Raf**. Animals were treated, data were analyzed and results are presented as described in the legend to Figure 1B. **A**. Ets and **B**. activated MEK/ERK.

We have shown previously [25] that the well-characterized MEK inhibitor U0126 suppressed the activated Ras Muv phenotype, but not the Muv phenotype conferred by loss of the Ets-like transcription factor. Therefore, as a control for pathway specificity, to ensure that MCP-mediated suppression of the Muv phenotype was not indirect, for example by inhibiting the cell cycle, we tested whether MCP compounds also inhibited the Muv phenotype of Ets animals. As expected, Ets animals were resistant to both MCP110 and MCP116, with no response at any dose (Figure 2A).

### MCP compounds act upstream of MEK

To determine the pathway level at which MCP compounds act, we continued our analysis with MEK/ERK [29]. Ectopic MEK/ERK is sufficient to drive excess vulval induction [29] and inhibition of MEK alone is sufficient to block the MEK/ERK-induced phenotype [25]. Thus, MEK/ERK animals should also be resistant to MCP compounds, which are believed to act by disrupting the upstream Ras/Raf interface. As predicted, MEK/ERK animals were also resistant to MCP110, MCP116 and MCP146 (Figure 2B), with no significant Muv differences observed in MCP- versus vehicle-treated animals. These results show that MCP110 and MCP116 target the Ras-Raf-MAPK pathway downstream of Ras and upstream of MEK.

### MCP compounds inhibit activated Raf

**Figure 3:**
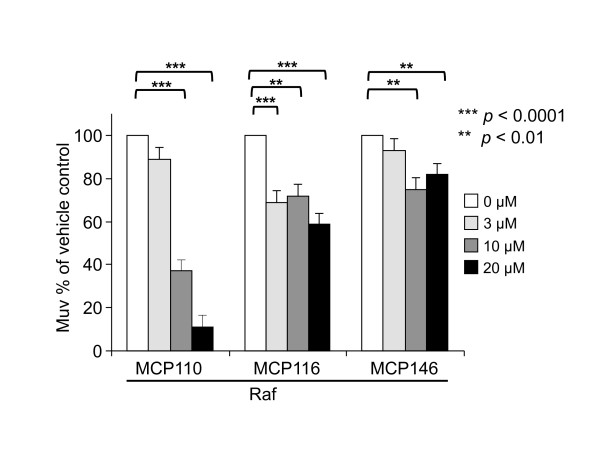
**MCP110 inhibits the Raf-AA Muv phenotype**. Animals expressing a constitutively activated Raf/LIN-45 protein were treated with vehicle (DMSO) or MCP110 as in Figure 1B. Bar graphs show the percentage of MCP-treated animals with a Muv phenotype, normalized to vehicle-treated animals. (***) and (**) indicate *p*-values of < 0.0001 and < 0.01, respectively.

To determine if MCP compound activity is due to blocking Raf, we compared the Muv phenotype of RafAA animals grown in the presence of MCP compounds or vehicle. Surprisingly, RafAA animals were sensitive to the action of MCP110, and, to a lesser extent, to MCP116 and the poorly active derivative MCP146 (Figure 3). Reversion of the Muv phenotype in these animals by MCP110 was dose-dependent and occurred with similar potency as in the Ras strain. This result suggested that MCP110 inhibition of Muv induction occurred at the level of Raf, rather than Ras. However, this Raf ortholog, although constitutively activated and sufficient to drive the Muv phenotype, still includes the Ras-interacting domains RBD and CRD [28]. Whether RafAA is completely Ras-independent is unclear [32,33]. It is possible that, in *C. elegans*, full activation of LIN-45AA requires a contribution from endogenous Ras. Further, given that MCP116 robustly inhibited Ras but not Raf induction of the Muv phenotype, it is possible that they do not bind in exactly the same manner to the Ras/Raf [Ras/RafAA] interface. We and others have attempted unsuccessfully to generate transgenic *C. elegans *strains carrying novel Raf/LIN-45 mutants predicted to be independent of Ras and lacking the Raf RBD and CRD. Transgenic animals harboring such constructs should definitively answer the questions of whether MCP110 reverts the Muv phenotype of RafAA-expressing animals by acting at the level of Ras or Raf, or whether MCP110 and MCP116 bind to Ras or Raf in the same manner. These questions will likely require structural information that is not yet available.

### MCP110 inhibits the physical interaction between Ras and Raf in mammalian cells

Another possibility to explain the ability of MCP110 to inhibit the Muv phenotype of RafAA-expressing animals is that this action does not occur as a consequence of disruption of the Ras/Raf interface. To confirm that MCP110 can in fact disrupt the Ras-Raf interaction, we turned to mammalian cells where biochemical analyses are more tractable.

The initial screening strategy for MCP compounds relied on the ability of the screened library components to separate the interaction of Ras and Raf in a yeast two-hybrid assay utilizing full-length versions of H-Ras and Raf-1 [18,20]. It remains unclear if the activity of MCP1, the originally identified MCP pharmacophore, relied on interaction with the Ras or the Raf protein interface. To address this question for MCP110, we took advantage of the fact that activated, GTP-bound Ras binds to Raf via interaction of its own effector domain (core residues 32-40 as well as flanking sequences) with the Ras binding domain (RBD) and cysteine-rich domain (CRD) of Raf. The affinity of the RBD of Raf for active Ras-GTP has been exploited to generate a widely used probe for this interaction, designated Raf-RBD, which is composed of residues 51-131 in the amino-terminal regulatory region of Raf-1. A GST-fusion protein of Raf-RBD [GST-Raf-RBD] has long been used as an affinity for pulldown assays to retrieve and quantitate the levels of activated Ras in cell lysates [34,35]. More recently, we have used the ability of Ras to recruit YFP- or GFP-tagged Raf-RBD as a visual probe for the subcellular localization of active Ras [36,37]. In each case, the readout is dependent on the physical interaction between Ras and Raf-RBD. Thus, to further understand the mechanism of action of MCP110 we analyzed its ability to disrupt the interaction between activated Ras and the Raf-RBD in a cell-based system.

**Figure 4:**
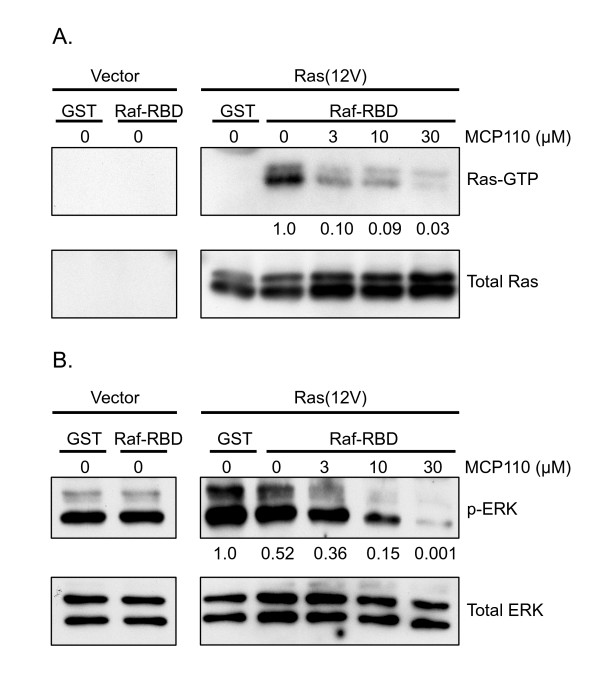
**MCP110 inhibits Ras/Raf interaction and signaling to ERK in mammalian cells**. *A. Pulldown assay for Ras-Raf interaction*. Pulldowns of active Ras bound to Raf-RBD were done using GST-Raf-RBD as described in Methods. NIH 3T3 cells were treated with vehicle or MCP110 (3, 10 and 30 μM) before and after lysis. Ras was detected by immunoblotting with anti-Ras antibody. Both Raf-RBD-bound Ras (Ras-GTP, upper panel) and total Ras in the lysates (lower panel) are shown. MCP110 disrupted the Ras/Raf interaction in a dose-dependent manner; numbers shown below panels indicate quantitation of Ras pulldown by densitometry, normalized to vehicle control. *B. Western blot analysis for active, phospho-ERK1/2*. The same lysates from cells depicted in panel A above were immunoblotted for phospho-ERK1/2 (P-ERK, upper panel) and for total ERK1/2 (total ERK, lower panel). Numbers shown below PERK panel indicate quantitation by densitometry, normalized to vehicle control. MCP110 decreased phospho-ERK1/2 in a dose-dependent manner.

We first performed pulldown assays in NIH 3T3 cells transiently transfected with both a constitutively active form of Ras and GST-Raf-RBD. Briefly, GST-Raf-RBD coupled to GSH-agarose beads was used to retrieve active Ras from lysates of cells treated with vehicle or MCP110 (see Methods), and the co-precipitated Ras was then detected by immunoblot analysis. We observed that the physical interaction between Ras and the Raf-RBD was disrupted by MCP110 in a dose-dependent manner, but not by the vehicle negative control (Fig. 4A, top panel). To confirm that less Ras was retrieved in the presence of MCP110 due to less effective interaction of Ras with the Raf-RBD rather than due to poor expression, we also assessed the total levels of Ras from equivalent amounts of lysates. We observed that Ras protein expression did not decrease upon MCP110 treatment (Fig. 4A, lower panel), indicating that Ras was still available for pulldown but was not retrieved.

If MCP110 decreased the physical interactions between Ras and Raf, it should also decrease downstream signaling through the Ras-Raf-MAPK pathway. We therefore examined the levels of phosphorylated ERK1/2 (p-ERK1/2) by immunoblotting with a phospho-specific antibody for ERK1/2 proteins that are phosphorylated at threonine 202 and tyrosine 204. Consistent with the dose-dependent inhibition of the Ras-Raf interaction, p-ERK levels were also reduced in a dose-dependent manner upon addition of MCP110 but not vehicle (Fig. 4B, upper panel), while the total levels of ERK remained unaffected (Fig. 4B, lower panel). Together, these results indicate that MCP110 can inhibit the physical interaction between Ras and Raf, as well as at least one functional consequence of that interaction, namely signaling to the downstream effector MAP kinases, ERK1/2. They also demonstrate that interaction of Ras with the Raf-RBD alone can be impaired by MCP110, consistent with the possibilities that the MCP110-mediated inhibition of the Muv phenotype induced in *C. elegans *by the LIN-45AA mutant Raf ortholog that retains the RBD may be due to MCP110 binding to either Ras/LET-60 or to Raf/LIN-45. These possibilites cannot be distinguished at present.

### MCP110 impairs localization of Raf-RBD to the plasma membrane in cells expressing constitutively active Ras

To corroborate our findings that MCP110 disrupted the physical interaction of Ras with the Raf-RBD, with a consequent functional impairment of downstream signaling, we wished to evaluate this interaction by another approach. As mentioned above, one biologically relevant method for doing so is to visually monitor the localization of a fluorescently tagged Raf-RBD. We have previously utilized Raf-RBD tagged with yellow fluorescent protein (YFP-Raf-RBD) to probe the subcellular localization of activated Ras [36]. We therefore used this probe in NIH 3T3 cells treated with vehicle or MCP110 to compare the localization of the Raf-RBD with that of a constitutively active, HA-tagged Ras, which was detected by Alexa-Fluor594-conjugated secondary antibody directed against the epitope tag. Cells were scored according to whether the YFP-Raf-RBD probe was localized to one of three major subcellular distributions: primarily membranes including plasma membrane; internal membranes and cytosol; or cytosol and nucleus.

**Figure 5:**
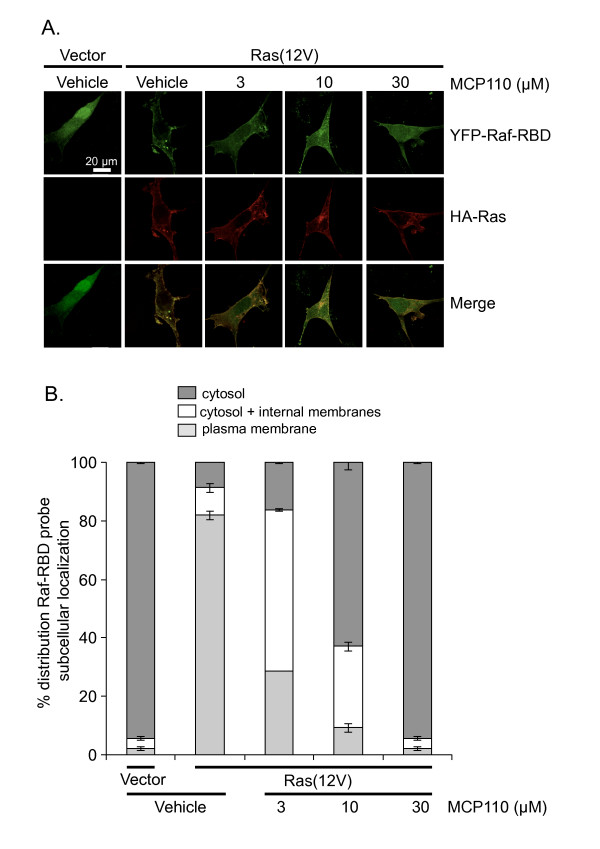
**MCP110 impairs recruitment of Raf-RBD to subcellular locations of active Ras**. *A. Recruitment of YFP-Raf-RBD to sites where Ras is localized, as detected by fluorescence microscopy*. NIH3T3 cells transiently expressing both HA-tagged active Ras and YFP-Raf-RBD were treated with vehicle or MCP110. Shown are representative images of cells quantitated in Panel B below. In the absence of active Ras (v.o.), YFP-Raf-RBD (green) was localized diffusely throughout the cytoplasm and nucleus, whereas in the presence of active Ras, the Raf-RBD probe was recruited to the plasma membrane and, like Ras (red), was nuclear-excluded ("vehicle" panels). Increasing concentrations of MCP110 increasingly shifted the YFP-Raf-RBD probe from the plasma membrane to the cytosol and to internal membranes and finally to both cytosol and nucleus, whereas Ras remained membrane-associated and nuclear-excluded. These results indicate dose-dependent disruption of the Ras/Raf-RBD interaction by MCP110. *B. Quantification of the distribution of YFP-Raf-RBD subcellular localization*. Cells treated and analyzed as described in Methods and depicted qualitatively in Panel C were binned according to whether the YFP-Raf-RBD probe accumulated primarily in the cytosol, or cytosol + internal membranes, or at the plasma membrane and was nuclear-excluded. MCP110 dose-dependently disrupts the ability of Ras to recruit Raf-RBD.

In cells expressing the YFP-Raf-RBD probe along with empty vector, YFP-Raf-RBD displayed a diffuse distribution throughout the cytosol and nucleus (representative images are shown in Fig. 5A and quantitation is shown in Fig. 5B). In stark contrast but as expected [36], co-expression of constitutively activated Ras resulted in exclusion of YFP-Raf-RBD from the nucleus and strong recruitment of Raf-RBD to membrane sites of Ras localization such as the plasma membrane and internal membrane compartments (Figs. 5A and 5B, vehicle treatment). Consistent with the ability of MCP110 to dose-dependently reduce the amount of Ras pulled down by GST-Raf-RBD (Fig. 4A, upper panel), it also dose-dependently impaired the recruitment of YFP-Raf-RBD to sites of activated Ras (Fig. 5A, MCP110 treatment). Indeed, with increasing doses of MCP110, YFP-Raf-RBD was restored to the cytosol and the nucleus (Fig. 5A, top row, and Fig. 5B, Ras + MCP110) even as the co-expressed active Ras remained membrane-bound and nuclear-excluded (Fig. 5A, middle row). These results are also consistent with MCP110 disruption of the physical interaction between Ras and Raf.

## Discussion

### MCP110 and MCP116 act downstream of Ras/LET-60 and upstream or at the level of Raf/LIN-45

Putative Ras-Raf interaction inhibitors such as MCP1 or derivatives based on MCP1, such as MCP110, have been shown previously to inhibit Ras-induced transcriptional reporter activity, cell migration, morphological and growth transformation as well as tumorigenicity in nude mice [20,21,23]. However, although these small molecules were originally identified via a yeast two-hybrid screen for inhibitors of interactions between H-Ras and Raf-1 [18-20], whether their biological activities in mammalian cells are due to physical disruption of this interaction has not been shown. Further, there have been conflicting reports in the literature as to whether the presence of mutationally activated and therefore Ras-independent B-Raf [B-Raf(V600E)] confers resistance to inhibition by MCP compounds [20,23]. However, these studies focused on different MCP analogs (MCP1 vs MCP110) and evaluated their actions in distinct B-Raf(V600E)-expressing melanoma lines, which could explain the different results. Therefore it was also not certain whether MCP compounds as a group act at the level of Ras or Raf.

Here we have used *C. elegans *as a genetic tool to investigate the level of the Ras-Raf-MAPK pathway at which MCP110 and its novel derivatives MCP116 and MCP146 act. The *C. elegans *orthologs are highly conserved with their mammalian counterparts, although the output of this pathway in C. elegans is vulval fate specification, and when hyperinduced leads to a Muv phenotype. We have shown here that MCP110 and MCP116 but not MCP146 inhibit the Muv phenotype driven by the *C. elegans *ortholog of Ras, but fail to inhibit the Muv phenotype driven by downstream elements of the pathway including MEK/MAPK and an Ets-like transcription factor. We have used this model previously to validate epistatically the actions of the MEK inhibitor U0126 [25,31], thus supporting the use of the Muv-driven phenotype caused by genetic lesions in elements of the Ras-Raf-MAPK pathway as a readout for pharmacological inhibition of the pathway. This phenotype has also been used previously by others to demonstrate specificity of first-generation farnesyltransferase inhibitors (FTIs) gliotoxin and manumycin, which target enzymatic modification of Ras, and thus can block the Ras Muv phenotype but not the Ets Muv phenotype [31].

Our analysis showed that MCP110 and MCP116 exerted activity against the Muv phenotype of constitutively activated RafAA animals, which was unexpected since RafAA had been reported to be Ras-independent and therefore should not be sensitive to disruption of the Ras-Raf interaction. Epistasis analysis involving these transgenes, in combination with loss-of-function mutations in upstream elements, suggested that their Muv phenotype is independent of Ras activity [32], but this was not proven conclusively since Ras/LET-60 itself was still present and functional. The mechanisms by which Raf and LIN-45 are activated have been extensively reviewed elsewhere [28,38,39], and it is clear that human and worm orthologs have similar regulatory mechanisms. However, the significant pharmacological inhibition of the activated Raf/LIN-45-driven Muv phenotype by MC110 seen hereimplies that the mechanisms by which LIN-45 is activated may still require Ras-Raf interaction. Indeed, this protein retains both the Ras-binding domain and CRD, thus leaving room for MCP action on the Ras-Raf interface. Our attempts, like those of many others in the *C. elegans *field, to generate more informative *lin-45 *transgenes were unsuccessful, so whether a truly Ras/LET-60-independent form of hyperactivated Raf/LIN-45 would be resistant to inhibition by MCP110 or MCP116 remains to be determined.

The discrepancies in previous observations of MCP activity leaves room to consider that the selectivity of these compounds for either Ras or Raf may rely in part on the model system or cell context. The ability of MCP110 and MCP116 to inhibit the Muv phenotype in animals expressing activated Ras/LET-60 may be due to the 86% identity of LET-60 shared with N-Ras in the first 164 amino acids, which allows LET-60 to possess all the biochemical functions of Ras proteins in mammals [40]. Conversely, some subtle isoform differences may also account for apparently discordant results between studies that do not evaluate precisely the same players [41].

While *C. elegans *does not replace mammalian cell culture models or higher organisms for *in vivo *studies, the use of a living organism for pharmacological studies, especially one like *C. elegans *that has been extensively characterized at the developmental and behavioral levels, can also lead to the detection of toxicity and off-target activity early in the drug discovery process, as well as to genetic identification of the target of unexpected biological activities [24]. The availability of additional transgenes for study will further increase the utility of this model system for drug discovery and development.

### MCP110 is a true protein-protein inhibitor of the Ras-Raf-RBD interaction

The original screen for MCP compounds, described in detail in [18,20], involved a modification of the yeast two-hybrid assay, which is a standard and powerful technique to detect protein-protein interactions and was the first method used to identify the interaction between Ras and Raf in live cells [42]. The technique, performed in this case in a hyperpermeable strain of yeast to enhance penetration of the cell wall by small molecules [18], detects these interactions by the transcriptional transactivation of a dual reporter system, and thus it is important for the interactions being analyzed to take place in the cell nucleus. Normally, Ras is post-translationally modified by farnesylation at its C-terminus for membrane targeting and biological activity, but in order to produce more functional Ras (bait) in the nucleus, the C-terminal modification motif was mutated to become insensitive to plasma membrane targeting [18]. The alteration of Ras localization to fit the purpose of the screen may have had an impact on the outcome, especially since it is thought that Raf interacts differently with farnesylated vs nonfarnesylated Ras proteins [43]. In addition, compounds registering positive in this screen may have had allosteric effects on regions of Raf not directly interacting with Ras.

To add another layer of complexity to the potential mechanism of action (MOA) of MCP110 and related compounds, as well as to experimental approaches to identifying inhibitors of Ras-Raf and to testing and validating inhibitor MOA, the activation of Raf-1 involves a series of steps involving membrane translocation, dephosphorylation at negative regulatory sites, and subsequent phosphorylation at activating sites in the kinase domain [44]. Activation of B-Raf is similar but not identical, and currently there is much attention being paid to possible influences of Raf-1 on B-Raf and vice versa [45-48]. Given that localization of Ras and the complex regulation of Raf are key determinants for activation of the signaling cascade, it is also possible that the original screen could have selected lead candidates affecting Ras or Raf interaction with other proteins that are positive regulators of the pathway. Several scaffolding proteins interact with members of the Ras-Raf-MAPK pathway to regulate the pathway by effects on protein localization or protein-protein interactions [49]. For example, SUR-8 is an evolutionarily conserved scaffold protein that is a positive regulator required for optimal Ras-MAPK signaling [50,51]. SUR-8 facilitates Ras-Raf complex formation [52], whereas reduction of SUR-8 suppresses activated Ras-mediated signaling in *C. elegans *[50,52]. The formation of a ternary complex of SUR-8 with activated Ras and Raf suggests that SUR-8 could also be a target of MCP110 activity, although the ability of MCP110 to impair the interaction of Ras with just the Raf-RBD interaction indicates that SUR-8 would not be an exclusive target.

An important finding of this study was the detection of MCP110-mediated disruption of the physical Ras-Raf interaction, providing evidence for the first time that MCP110 significantly disrupts the protein-protein interface involving full-length H-Ras and the Raf-RBD in mammalian cells. This indicates that MCP110 can act as a true PPI inhibitor. Whether it also disrupts the interaction of K-Ras and N-Ras with Raf-RBD remains to be determined. Also remaining to be determined is whether it shows selectivity for disruption of interactions of Ras with the different Raf isoforms. The strong association of Raf-RBD with Ras-GTP versus Ras-GDP [53] supports MCP110 disruption of the Ras-Raf complex, but the selectivity of MCP110 to disrupt interactions between Ras and Raf versus other GTPase/RBD pairs has also not yet been determined. Given that it is presently unclear whether MCP110 binds to Ras, to Raf-RBD or both, it would also be of interest to evaluate the ability of MCP110 to disrupt the interaction of the RBDs of other GTPases and their effectors. For example, RBDs of Ral GEFs (RalGDS, Rgl1-3) can interact with Ras as well with the Rap1A GTPase [54-56]. Whether MCP110 can also disrupt the interaction of Ras with RalGEF RBDs or Rap1A with Raf-RBD will be important to determine. Additionally, the effector domain of the Ras-related GTPase Rit provides a similar surface to that of Ras [41], and may thus also be disruptable by MCP110, MCP116 or related compounds. Finally, why MCP110 and MCP116 did not display the same ability to inhibit the RafAA Muv phenotype is currently unclear. The availability of structural information on complexes of MCP110 and of MCP116 with Ras-Raf would be of great assistance in making predictions about the most fruitful avenues to pursue in these directions.

## Conclusions

Here we used both mammalian cell culture studies and the genetically tractable *C. elegans in vivo *model to investigate the activity of putative Ras/Raf interaction inhibitors. We dissected the pathway and were able to determine that MCP compounds act downstream of Ras/LET-60 and upstream or at the level of Raf/LIN-45, thereby providing additional proof-of-principle for the use of *C. elegans *as a simple and attractive model for the characterization of novel or already isolated Ras pathway inhibitors. The work presented here has contributed to a better understanding of the mechanism of action of putative Ras/Raf interaction inhibitors based on the MCP110 pharmacophore. In addition to supporting previous conclusions that MCP110 significantly inhibits the signals caused by activated Ras *in vitro *and *in vivo*, we have been able to narrow the requirements for its activity by successfully using it to disrupt the Ras/Raf-RBD interaction. Given these results, it will be interesting to see if more focused screens involving Ras and the Raf-RBD can identify additional potent and selective Ras/Raf interaction inhibitors.

## Methods

### *C. elegans *strain maintenance and culturing conditions

Strain maintenance and nomenclature are as described [57,58]. Strains were cultured on 2% NG agar plates seeded with *E. coli *strain OP50. SD418 *gaIs37 *(*mek-2*(gf)*+mpk-1*(gf))strain was maintained at 15°C and switched to 25°C to induce its conditional hyper-induced phenotype.

Briefly, activated Ras results from an *in situ *mutation in LET-60/Ras that causes a G13E change equivalent to G13E in human Ras, which is functionally similar to the well known G12V activating mutation [27]. Activation of Raf is a multistep process in which several regulatory residues are modified to regulate its kinase activity. Transgenic alteration of the conserved Akt negative regulatory sites from serine to alanine at residues 312 and 453 ("AA") in Raf (RafAA) leads to a hyper-induced phenotype comparable to that conferred by activated Ras [28,33]. Activated MEK/ERK results from transgenic expression of both activated *Drosophila *MEK and activated *C. elegans *ERK (MPK-1), all driven by a heat-shock promoter. Consequently, the MEK/ERK Muv phenotype is temperature-sensitive, such that animals are grown at 25°C to induce a Muv phenotype, but are wild type at 15°C [29]. Finally, "Ets" refers to loss of the LIN-1/Ets transcription factor function. LIN-1 inhibits vulval fate, so LIN-1 loss results in hyper-induction [30].

### Drug assays and quantification of the multivulva (Muv) phenotype

We have described in detail the experimental procedures for *C. elegans *drug treatments and phenotype quantification [25]. Briefly, experiments were performed in 6-well tissue culture plates in which only the four corner wells were filled with 3 ml of 2% NG agar. Either vehicle alone (dimethylsulfoxide; DMSO) or vehicle plus experimental drug (MCP110, MCP116, or MCP146) was diluted in M9 buffer and applied in a defined volume to the agar in each well to achieve the final dose. Plates absorbed the drug overnight, then were seeded with 90 μl of OP50 overnight culture and allowed to grow for 24 hours to ensure a suitable bacterial lawn.

To obtain a population of treated animals that was developmentally synchronous, we harvested embryos during a narrow time frame. For each strain and drug, 12-15 adult hermaphrodites laid eggs for 3 hours, after which the parents were removed.

Animals to be assayed were exposed to drug throughout development. Animals harboring activated Ras, Raf and MEK/MAPK were scored as early adults using the dissecting microscope. Animals harboring activation of the Ets-like transcription factor were scored at the 4^th ^larval stage (L4) because adult pseudovulvae were too distorted to quantify clearly [25]. For DIC microscopy, animals were mounted on slides in M9 buffer containing 5 mM sodium azide.

To reproducibly score the outcome of drug assays, we used a specific set of phenotypic criteria. First, we categorized animals in a binary assay as Muv or non-Muv, depending on the presence of the ectopic pseudovulvae that indicate hyper-induction of vulval tissue. Second, we quantified the number of ectopic pseudovulvae. Each genotype assayed had a different baseline for degree of hyper-induction, and therefore for each genotype the baseline was re-established such that animals treated with the experimental drug were normalized to the level of hyper-induction in animals treated with vehicle.

### Statistical analyses

In order to assess the overall effects of strain, concentration, and drug, we used a three-way ANOVA model with fixed main effects for strain, concentration and drug, all pairwise and three-way interactions to compare the mean proportions of standardized MUV phenotype scores. The three-way interaction was determined to be statistically significant at the 0.05 level (p < 0.0001), supporting the significance of the dose-dependent effects of MCP compounds that we observed in Ras/LET-60 and Raf/LIN-45 animals.

### Cell culture and transfections

NIH 3T3 mouse fibroblasts were grown in DMEM (Sigma-Aldrich, St. Louis, MO) supplemented with 10% GCS calf serum (GIBCO/Invitrogen, Carlsbad, CA) and 1% penicillin/streptomycin and maintained in 5% CO_2 _at 37°C. Cells were plated the day before transfection at a density of 200,000 cells per 60 mm dish or 100,000 cells per 35 mm dish (or in a 6-well plate), for the pulldown and co-localization assays, respectively. For pulldown assays, pcDNA3.1 (vector only, v.o.) or pcDNA3.1 encoding activated H-Ras(12 V) were transfected transiently into cells using *Trans*IT-LT1 Transfection reagent (Mirus, Madison, WI) according to the manufacturer's specifications. For co-localization assays, pEYFP-Raf-RBD was cotransfected with either empty pCGN-HA vector (v.o.) or pCGN-HA encoding activated H-Ras(12 V). Immediately after transfection, a designated amount of either DMSO vehicle or MCP110, MCP116 or MCP146 was added at 3, 10 or 30 μM and further assays were performed after 48 h.

### Pulldown assays and immunoblotting

Transiently transfected NIH 3T3 cells (see above) were lysed in 400 μl of freshly prepared Magnesium Lysis Buffer (MLB) combined with protease inhibitor cocktail (BD BaculoGold, BD Biosciences Pharmingen, San Jose, CA). Lysates were cleared by centrifugation at 12,000 rpm for 10 min at 4°C, and protein concentration was measured in a Bradford Assay (Bio-Rad Laboratories, Hercules, CA). GST fusion proteins of the Raf-1 Ras binding domain (GST-Raf-RBD) were prepared from pGEX-2T encoding Raf-RBD as described previously [35,59]. Empty vector pGEX-2T plasmid encoding GST alone was a negative control. A total of 100 μg of MCP110 pre-treated protein lysate was incubated with 10 μl of glutathione agarose beads (Sigma) previously coupled to GST alone or to GST-Raf-RBD. Parallel to the incubation of lysate with beads, additional drug was added and the pulldown reaction was performed in a final volume of 500 μL, rocking for 1 h at 4°C. Proteins bound to beads were collected, washed three times in lysis buffer and eluted in non-reducing protein sample buffer. Pulldown samples and total protein were analyzed by SDS-polyacrylamide gel electrophoresis and western blotting.

For immunoblotting, membranes were blocked in 5% non-fat dry milk and incubated overnight with primary antibodies diluted to 1:3,000 for H-Ras (146, Quality Biotech, NJ), 1:500 for p-ERK (Cell Signaling Technology, Danvers, MA) or 1:2,000 total ERK (Cell Signaling Technology) dilution, overnight at 4°C. Membranes were washed and incubated for 1 h in a 1:30,000 dilution of anti-mouse or anti-rabbit IgG-horseradish peroxidase antibody (GE Healthcare/Amersham, Piscataway, NJ), washed extensively with TBS-T and developed with SuperSignal West Dura Extended Duration substrate (Thermo Scientific, Rockford, IL).

### Inmunofluorescence assay for Ras recruitment of Raf-RBD probe

NIH 3T3 cells were grown on coverslips, transiently transfected as above, and treated with MCP110 for 48 h before fixation in 3.7% formaldehyde, permeabilization with 0.5%Triton and blocking in 2% BSA for 1 h at room temperature. Coverslips carrying fixed cells were incubated in a 1:200 dilution of anti-HA antibody (Covance, Emeryville, CA) for 1 h, followed by two washes in 1× Phosphate Buffered Saline (PBS), an additional incubation with a 1:1,000 dilution of AlexaFluor594 anti-mouse conjugated secondary antibody (Molecular Probes, Eugene, OR) for 30 min and washed three times with 1× PBS. Coverslips were mounted into a glass microslide with ProLong Gold antifade (Invitrogen/Molecular Probes, Eugene, OR) mounting medium and cells were visualized by confocal microscopy.

## List of abbreviations

ATP: adenosine triphosphate; BSA: bovine serum albumin; CRD: cysteine rich domain; DMEM: Dulbecco's modified Eagle medium; DMSO: dimethyl sulfoxide; EGF: extracellular growth factor; ERK: extracellular signal-regulated kinase; FTI: farnesyltransferase inhibitor; GST: glutathione S-transferase; GTP: guanosine triphosphate; MAPK: mitogen-activated protein kinase; MCP: Morphochem product; MLB: magnesium lysis buffer; MOA: mechanism of action; Muv: multivulva; NG agar: neutral growth agar; PBS: phosphate buffered saline; PI3K: phosphatidylinositol 3-kinase; PPI: protein-protein interaction; RBD: Ras binding domain; SDS: sodium dodecyl sulfate; VEGFR: vascular endothelial growth factor receptor; WT: wild type; YFP: yellow fluorescent protein.

## Competing interests

VK is a full time employee and a shareholder of NexusPharma, Inc.

## Authors' contributions

VGP carried out the experiments and drafted the manuscript. DJR contributed to experimental design and interpretation of studies involving *C. elegans *and assisted in drafting the manuscript. JKA captured the images and scored the subcellular localization of the YFP-Raf-RBD probe. CM and LJE contributed to the design of *C. elegans *data collection and performed and supervised, respectively, statistical analyses of the data. VK provided MCP compounds and contributed to experimental design and interpretation. CJD and ADC conceived of the study, and contributed to study design and interpretation and to preparation of manuscript and figures. All authors read and approved the final manuscript.
